# Identification of sulfur metabolism-related gene signature in osteoarthritis and TM9SF2’s sustenance effect on M2 macrophages' phagocytic activity

**DOI:** 10.1186/s13018-023-04384-2

**Published:** 2024-01-13

**Authors:** Shuang Zheng, Yetian Li, Li Yin, Ming Lu

**Affiliations:** 1 Department of Rheumatology, The First Affiliated Hospital of Anhui Medical University, No.218 Ji Xi Road, Hefei, 230032 Anhui China; 2 Department of Orthopedics, The First Affiliated Hospital of Anhui Medical University, No.218 Ji Xi Road, Hefei, 230032 Anhui China

**Keywords:** Osteoarthritis, Sulfur metabolism, Diagnosis, TM9SF2, Phagocytosis

## Abstract

**Background:**

Osteoarthritis (OA) is a chronic and low-grade inflammatory disease associated with metabolism disorder and multiple cell death types in the synovial tissues. Sulfur metabolism has not been studied in OA.

**Methods:**

First, we calculated the single sample gene set enrichment analysis score of sulfur metabolism-associated annotations (i.e., cysteine metabolism process, regulation of sulfur metabolism process, and disulfidptosis) between healthy and synovial samples from patients with OA. Sulfur metabolism-related differentially expressed genes (DEGs) were analyzed in OA. Least absolute shrinkage and selection operator COX regression were used to identify the sulfur metabolism-associated gene signature for diagnosing OA. Correlation and immune cell deconvolution analyses were used to explore the correlated functions and cell specificity of the signature gene, *TM9SF2*. TM9SF2’s effect on the phagocytosis of macrophages M2 was analyzed by coculturing macrophages with IgG-coated beads or apoptotic Jurkat cells.

**Results:**

A diagnostic six gene signature (i.e., *MTHFD1, PDK4*, *TM9SF2*, *POU4F1*, *HOXA2*, *NCKAP1*) was identified based on the ten DEGs, validated using GSE12021 and GSE1919 datasets. *TM9SF2* was upregulated in the synovial tissues of OA at both mRNA and protein levels. The relationship between TM9SF2 and several functional annotations, such as antigen processing and presentation, lysosome, phagosome, Fcγ-mediated phagocytosis, and tyrosine metabolism, was identified. *TM9SF2* and macrophages M2 were significantly correlated. After silencing *TM9SF2* in THP-1-derived macrophages M2, a significantly reduced phagocytosis and attenuated activation of PLC-γ1 were observed.

**Conclusion:**

A sulfur metabolism-associated six-gene signature for OA diagnosis was constructed and upregulation of the phagocytosis-associated gene, *TM9SF2*, was identified. The findings are expected to deepen our understanding of the molecular mechanism underlying OA development and be used as potential therapeutic targets.

**Supplementary Information:**

The online version contains supplementary material available at 10.1186/s13018-023-04384-2.

## Introduction

Osteoarthritis (OA) is the most prevalent degenerative joint disease. OA leads to chronic low-grade synovitis, joint pain, and even disability [1, 2]. Synovial tissues include a thin membrane lining the inside of synovial joints, comprising immune cells (e.g., monocytes, macrophages, dendritic cells), fibroblasts, and a sub-lining vascularized connective tissue. Among multiple immune cell types in OA synovium, macrophages are the most common immune cells in inflammatory synovial tissues and are correlated with the disease’s clinical symptoms [3–6]. Notably, OA is always first diagnosed at an irreversible stage after bone damage has occurred [7]. The diagnosis by testing early or pre-osteoarthritic changes before the onset of irreversible changes is crucial for understanding its underlying pathogenesis and designing treatment strategies. Metabolic changes in synovial tissues may represent the earliest measurable changes and are considered reversible [7, 8]. Sulfur-containing amino acids play an important role in health and some inflammatory diseases, such as ulcerative colitis [9–11]. Cysteine is a semi-essential proteinogenic amino acid given its synthesis from methionine and serine by trans-sulfuration. L-cysteine exerts an anti-inflammatory effect, including enhancing the inhibitory effect of vitamin D under oxidative stress [12–15]. To date, sulfur metabolism in OA has not been studied.

In response to the low-grade inflammation stress in OA, multiple forms of cell death occur in OA synovial tissues, such as apoptosis and ferroptosis [16, 17]. Notably, SLC7A11, coding for the sodium-independent cystine-glutamate antiporter Xc- [18], is involved in the clearance of apoptotic cells [19] and inhibition of cellular ferroptosis [20, 21]. SLC7A11 inhibition can increase efferocytosis of dendritic cells blocking the cysteine transport into cells. Interestingly, a recent study found that SLC7A11 was involved in regulating a new form of cell death, disulfidptosis, by mediating cysteine intake and inhibiting ferroptosis under glucose starvation conditions [18, 22]. Disulfidptosis is induced by aberrant disulfide bonds in actin cytoskeleton proteins and F-actin collapse and can be promoted by activating cytoskeleton-associated WAVE regulatory complex and Rac. In addition to disulfidptosis, SLC7A11 is also involved in two other sulfur-associated functions as per the gene ontology (GO), namely, “regulation of sulfur metabolic process” and “cysteine metabolic process” [23, 24]. Among the top-ranked proteins that can inhibit disulfidptosis in SLC7A11^high^ cells, GYS1, NDUFA11, NUBPL, and LRPPRC promote glycogen synthesis and mitochondrial oxidative phosphorylation, suggesting the close relationship between energy metabolism and disulfidptosis [22]. Therefore, whether the expression of sulfur metabolism-related genes is enhanced or weakened in OA synovial tissues merits investigation.

To explore the role of sulfur metabolism-associated genes in OA, including those involved in cysteine metabolism, sulfur metabolism, and disulfidptosis, differentially expressed genes among them were identified and a consensus cluster analysis was performed to compare the C1/C2 groups and OA/healthy groups. We constructed a gene signature using LASSO COX regression and validated their diagnostic efficiency using receiver operating characteristic curve (ROC) analysis, followed by external validation using the expressional data from GSE12021 and GSE1919. To gain a deeper understanding of the relevant underlying molecular mechanisms, immunological relevance, and cell specificity of the signature gene, *TM9SF2*, we conducted correlational, functional enrichment, and immune cell deconvolution analyses. After silencing the expression of *TM9SF2* in THP-1-derived macrophages, phagocytosis by macrophages M2 was weakened. Our findings can provide molecular clues for the role of sulfur metabolism in the pathogenesis of OA along with new therapeutic targets.

## Methods and materials

### Data resource and processing

Microarray and single-cell RNA sequencing (scRNA-seq) data were obtained from the gene expression omnibus (GEO) database (https://www.ncbi.nlm.nih.gov/geo/query/acc.cgi?acc=) with accession IDs, GSE55235/GSE55457/GSE82107/GSE12021/GSE1919, using the ‘GEOquery’ R package [25]. The platform annotation documents were downloaded from GEO and annotated for microarray probes by using the ‘merge’ R command after Log_2_(*x* + 1) normalization. Batch effects among datasets GSE55235 (10 healthy and 10 OA synovial tissues), GSE55457 (10 healthy and 10 OA synovial tissues), and GSE82107 (7 healthy and 10 OA synovial tissues) were removed by the command, ‘removeBatchEffect(data, batch = datasets, design = group)’ in ‘Limma’ R package [26]. Nine healthy and 10 OA synovial tissues in the GSE12021 dataset and 5 healthy and 5 OA synovial tissues in the GSE1919 dataset were obtained.

### Single-sample gene set enrichment analysis (ssGSEA)

ssGSEA was performed and the enrichment score of each sample was calculated for energy metabolism- and sulfur metabolism-associated gene sets using the ‘GSVA’ R package [27]. Boxplots and heatmaps were drawn using the ‘ggplot2’ R package [28].

### Differentially expressed gene (DEG) analysis

Student’s *t*-test was used to assess the significance of DEGs between OA and healthy groups. The ‘Limma’ R package was used to identify DEGs between C1 and C2 classes. Briefly, the ‘lmFit’ function for multiple linear regression was used, followed by the ‘eBays’ function to calculate moderated t-statistics/F-statistics and log odds of differential expression by using empirical Bayes moderation of the standard errors toward a common value, and finally, statistically significant DEGs were obtained.

### Cluster analysis

Consensus cluster analysis was performed using the ‘ConsensusClusterPlus’ R package [29] for the 10 sulfur-related DEGs. The agglomerative partition around medoids (PAM) clustering with a 1-Pearson correlation distance and resampling of 80% of the samples in 10 repetitions was used in the analysis. Clustering by tSNE was performed for the merged dataset, GSE55235/GSE55457/GSE82107, using the ‘Rtsne’ R package [30]. Specifically, we first obtained the z-score of the expression profile and used the ‘Rtsne’ function for dimensionality reduction analysis to obtain the dimensionality-reduced matrix.

### Functional enrichment analysis

GO and pathway analysis based on gene cluster GO terms [including biological process (BP), molecular functions (MF), and cellular components (CC)] and Kyoto Encyclopedia of Genes and Genomes (KEGG) annotations were obtained by using the ‘clusterProfiler’ R package [31] and virtualized by the ‘ggplot2’ R package. GSEA was performed by pre-ranking genes based on their correlation with classes C1/C2. We subsequently performed GSEA by pre-ranking the c2.cp.kegg.v7.4.symbols.gmt gene set from MsigDB [32, 33] by using the ‘clusterProfiler’ R package.

### Immune cell deconvolution analysis

Immune cell deconvolution analysis methods, CIBERSORT [34] and xCell [35] were used to calculate the proportion and score of immune cell infiltration, respectively, in the synovial microenvironment using the ‘IOBR’ R package [36]. Boxplots of proportions and scores were drawn with the ‘ggplot2’ R package. Student’s t-test was performed to compare between groups.

### Least absolute shrinkage and selection operator (LASSO)-COX regression analysis

LASSO-COX regression analysis was performed using the ‘glmnet’ R package to identify signature genes and their coefficients to predict the risk of OA [37, 38].

### ROC analysis

We used the ‘pROC’ R package [39] to perform ROC analysis, and the results were visualized with the ‘ggplot2’ R package.

### Correlational analysis

Correlational analysis was performed by computing Pearson correlation coefficients between the expression levels of two genes or between the expression level of gene A and the infiltrated level of immune cell B, using the function, ‘cor’, of the base R package.

### Phagocytosis analysis

THP-1-derived macrophages M2 were induced with 20 ng/ml PMA for two days followed by polarization with 20 ng/ml IFN-r and 100 ng/ml LPS for another two days. The macrophages were co-cultured with IgG-PE labeled latex beads (Phagocytosis Assay Kit (Cat. 600540; Cayman Chemical, Ann Arbor, MI, USA)) or PKH26-labeled apoptotic Jurkat cells for phagocytosis or efferocytosis for 1 h, respectively. For efferocytosis, apoptosis was induced in PKH26-labeled Jurkat cells by UV irradiation for 15 min. After apoptosis induction in Jurkat cells, they were added to the macrophage culture in a ratio of 1:10 (macrophages to Jurkat cells). The efficiency of phagocytosis/efferocytosis was quantified by flow cytometry.

### Interaction among proteins

The protein–protein interaction (PPI) network analysis was performed using the STRING database (https://cn.string-db.org), and the Analysis Tab on the website was used for conducting KEGG pathway analysis for the PPI network. Potential interactors of TM9SF2 were identified using BioGRID (https://thebiogrid.org) and HIPPIE (http://cbdm-01.zdv.uni-mainz.de/~mschaefer/hippie/index.php). TM9SF2 was knocked down by shRNA and its mRNA and protein levels were quantified by qPCR and western blotting (#PA-48517, ThermoFisher), respectively. PLC-γ1 (#2822, CST) activity was assessed based on Y783 phosphorylation (#2821, CST) by western blotting.

## Results

### Scores of sulfur-related annotations are reduced in the osteoarthritic synovium

**Fig. 1:**
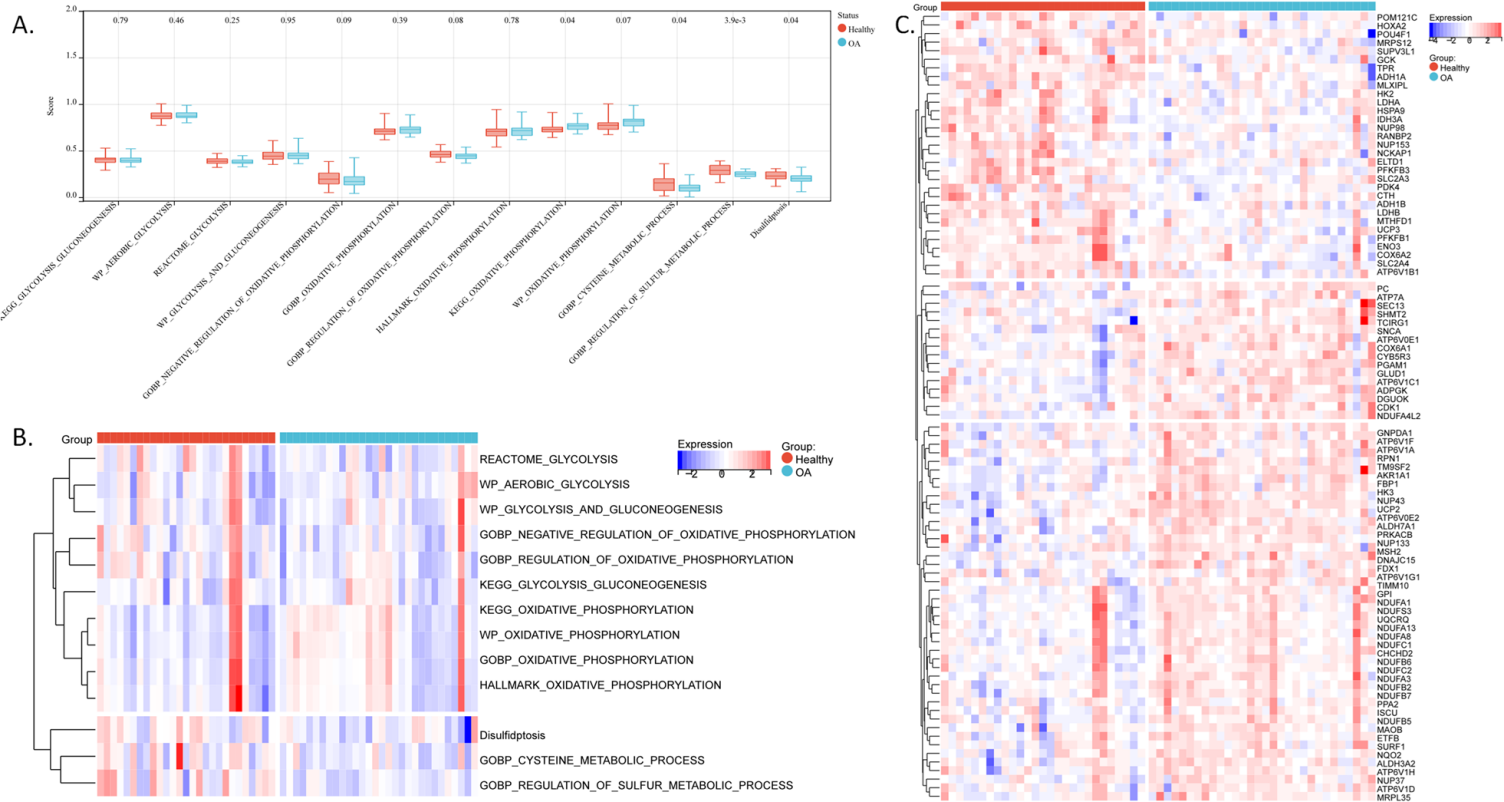
ssGSEA scores for different gene sets related to glycolysis, OXPHOS, cysteine metabolic process, sulfur metabolic process, and disulfidptosis in synovial samples. **A** Boxplot shows the ssGSEA score for each gene set in synovial samples. Only the scores of the cysteine metabolic process, regulation of the sulfur metabolic process, and disulfidptosis gene sets are significantly downregulated. The *P* value is shown at the top of the figure for each group. **B** The heatmap shows the normalized ssGSEA score for each gene set. **C** The heatmap shows the expression of DEGs between healthy (red samples) and OA (light blue samples) groups. DEGs: differentially expressed genes between OA and healthy groups

We first merged the synovial tissue microarray datasets, GSE55235 (10 healthy/10 OA), GSE55457 (10 healthy/10 OA), and GSE82107 (7 healthy/10 OA). We downloaded gene sets (Additional file 1) related to energy and sulfur metabolism annotations from MSigDB along with the gene set regulating cell disulfidptosis, particularly SLC7A11, from the literature [22]. Based on the expression matrix, by ssGSEA, we analyzed energy metabolism (i.e., glycolysis, oxidative phosphorylation) and sulfur metabolism (i.e., sulfur metabolism, cysteine metabolism, and disulfidptosis) in osteoarthritic synovial tissues compared with those in normal synovial tissues (Fig. 1A,B). The scores for sulfur metabolism, cysteine metabolism, and disulfidptosis were significantly reduced in OA tissues (Fig. 1A,B), suggesting their potential inhibitory effect on the pathogenesis of OA or reduction in sulfur metabolism with OA progression. The scores of glycolysis and oxidative phosphorylation (OXPHOS) were all unchanged in OA tissues, except for the “KEGG_OXIDATIVE_PHOSPHORYLATION” score in OA, which was increased (Fig. 1A,B), further indicating increased OXPHOS in osteoarthritic synovial tissues. Furthermore, between the healthy and OA groups, we obtained 91 DEGs by t-test (Fig. 1C).

### Classification of synovial tissues based on sulfur metabolism-associated DEGs and their potential molecular mechanisms of action

**Fig. 2:**
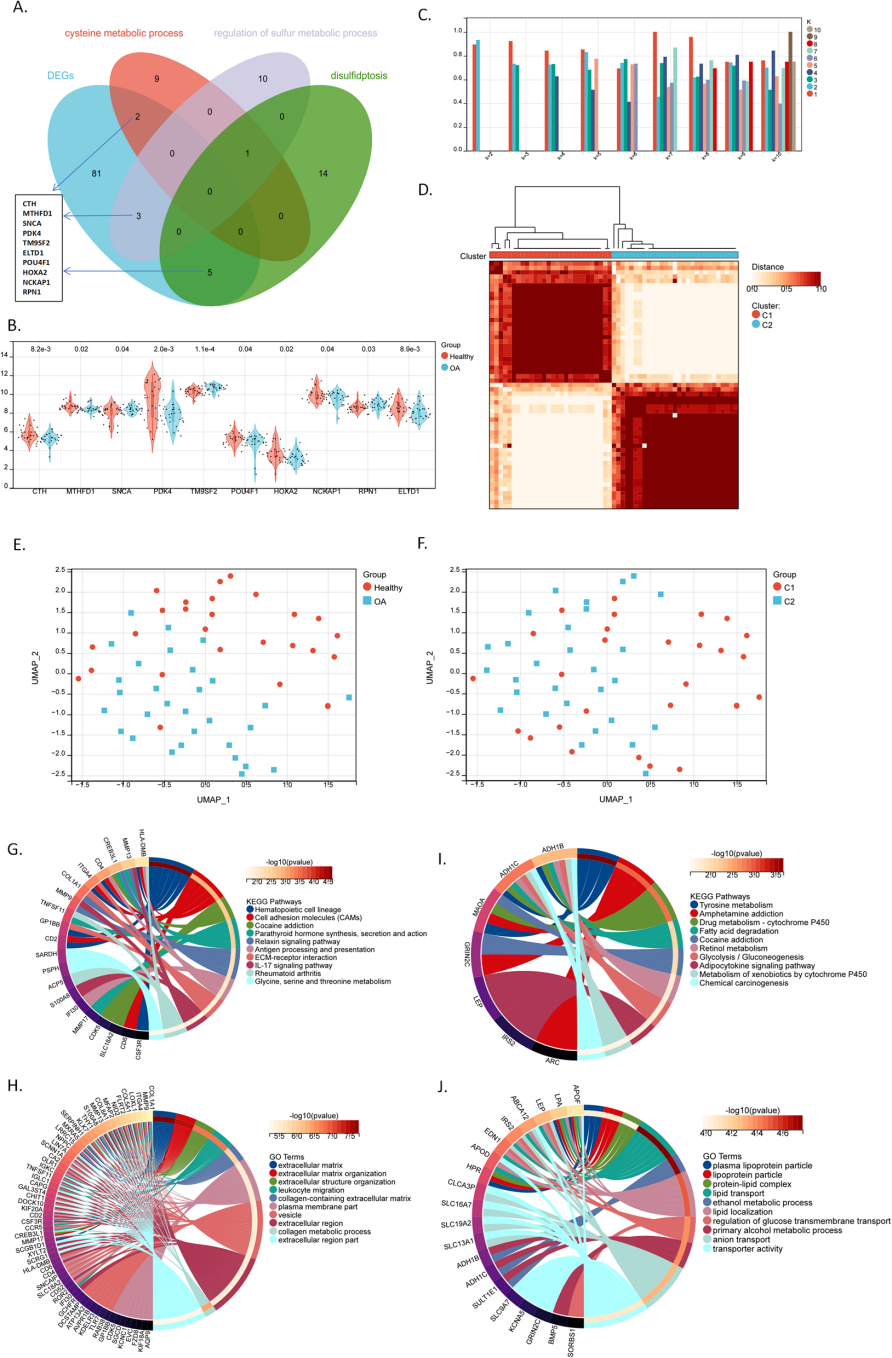
Sulfur-associated DEGs distinguish classes C1 and C2, along with their differentially enriched functions. **A** The Venn diagram shows ten overlapping genes between DEGs and sulfur-associated gene sets (i.e., cysteine metabolic process, regulation of sulfur metabolic process, and disulfidptosis gene sets). DEGs: differentially expressed genes between OA and healthy groups. **B** The violin plot shows the expression of the ten 10 sulfur metabolism-associated DEGs. **C** The bar graph shows the consistency within each group following consensus cluster analysis. Different colors represent the K-value or the number of groups set in each round of the clustering algorithm. The clustering solution based on K = 2 groups shows a higher intra-group consistency compared to other K values. **D** The heatmap shows two classes distinguished by consensus cluster analysis based on the 10 sulfur-associated DEGs. **E**, **F** Clusters identified by tSNE analysis for the expression profile of all genes. These are labeled with colors for healthy/OA groups (**E**) or C1/C2 groups (**F**), and the results suggest a consistency between the two classifications **G**, **H**. KEGG (**G**) and GO (**H**) enriched functions for the upregulated genes in class C2 (OA-dominated); e.g., cell adhesion molecules, IL-17 signaling pathways, rheumatoid arthritis, extracellular matrix, and leukocyte migration. **I**, **J** KEGG (**I**) and GO (**J**) enriched functions for genes upregulated in class C1 (healthy-dominated); e.g., tyrosine metabolism, fatty acid degradation, and lipid transport.`

To select characteristic genes in the OA synovium among the 91 DEGs, we overlapped the 91 DEGs with sulfur metabolism-associated genes (Fig. 2A). We obtained 10 DEGs from sulfur metabolism-associated annotations, including *CTH*, *MTHFD1*, *SNCA*, *PDK4*, *TM9SF2*, *ELTD1*, *POU4F1*, *HOXA2*, *NCKAP1*, and *RPN1* (Fig. 2B). To understand their ability to distinguish between OA and healthy groups, we performed a consensus cluster analysis according to the expressional level of the ten sulfur metabolism-associated genes (Fig. 2C,D). The ten-gene classified C1 and C2 groups were consistent for the OA and healthy groups (Fig. 2E,F).

To explore the potential functions of the ten sulfur metabolism-associated genes, we performed a functional enrichment analysis by using all DEGs between C1 and C2. The upregulated genes in the OA-dominated C2 group were mainly enriched in the following KEGG pathways: “Hematopoietic cell lineage,” “Cell adhesion molecules (CAMs),” and “Antigen processing and presentation” (Fig. 2G), with GO annotations of “extracellular matrix,” “leukocyte migration,” and “vesicle” (Fig. 2H). The upregulated genes in the healthy samples-dominated C1 group were primarily enriched in the KEGG pathways of “Tyrosine metabolism” and “Fatty-acid degradation” (Fig. 2I), with GO annotations of “lipid transport”, “ethanol metabolic process”, “lipid localization”, and “regulation of glucose transmembrane transport” (Fig. 2J). These suggest the potential associations between sulfur metabolism and extracellular matrix, antigen processing/presentation, tyrosine metabolism, and lipid/glucose transport.

### Identifying a sulfur metabolism-associated gene signature for diagnosing OA

**Fig. 3:**
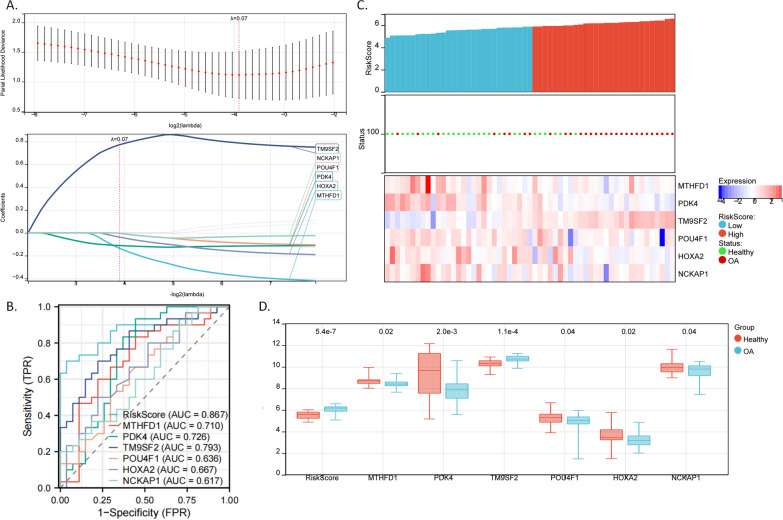
Establishment of the diagnostic gene signature by LASSO COX regression analysis. **A** LASSO COX regression for 9 sulfur-associated genes. The coefficient profile plot was generated against the log (lambda) sequence (Upper). LASSO coefficient profiles of the nine genes in the merged dataset (Lower). **B** Accuracy of the diagnostic model for the six-gene signature to predict OA diagnosis, as evidenced by the receiver operating characteristic (ROC) curve analysis. When AUC (i.e., Area under the ROC curve) is 0.5, it means there is a 50% chance that the model can distinguish between positive and negative classes; 0.7 ≥ AUC > 0.6: acceptable discrimination; AUC > 0.7: excellent discrimination. **C** Detailed diagnostic information (healthy/OA) and expressional patterns of candidate genes differ between high-risk score and low-risk score groups. Upper layer: level of risk score for each sample; middle layer: OA status (red: OA, green: healthy); lower layer: heatmap shows the gene expression. **D** The boxplot shows differential expression levels of the six genes between healthy and OA groups

We performed a LASSO COX regression analysis for the nine genes (since we planned to use the GSE12021 and GSE1919 datasets to verify the diagnostic efficacy of gene biomarkers, we removed *ELTD1*, which was absent in the two datasets) and identified the diagnostic gene signature comprising six genes (Fig. 3A). The risk score could be used for diagnosing OA. The following was the calculation: Risk Score =  − 0.1355 × *MTHFD1* (expression level) + (− 0.1114) × *PDK4* + 0.7726 × *TM9SF2* + (− 0.0031) × *POU4F1* + (− 0.0374) × *HOXA2* + (− 0.0047) × *NCKAP1*. The diagnostic ROC of the risk score showed excellent predictive power (area under the ROC curve (AUC) = 0.867, Fig. 3B). From the heatmap (Fig. 3C) and boxplot (Fig. 3D) of gene expression, we observed that most of the high-risk group samples were in the OA group with *TM9SF2* being significantly upregulated while the other five genes were significantly downregulated. Most of the high-risk group samples were of OA (Fig. 3C).

**Fig. 4:**
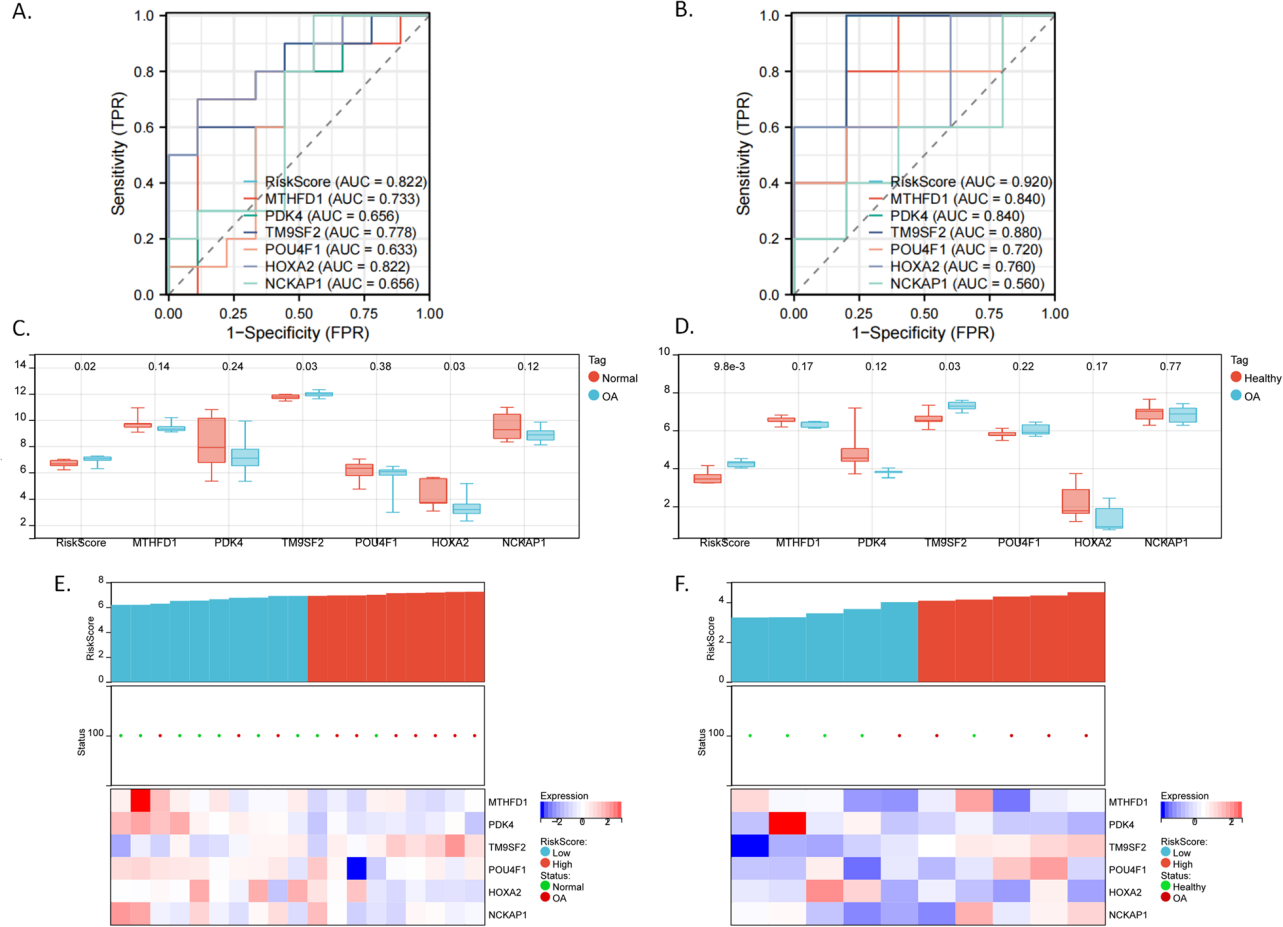
Validation of the diagnostic six-gene signature in GSE12021 and GSE1919 datasets. **A**, **B** Accuracy of the diagnostic model for the six-gene signature in predicting osteoarthritis by ROC analysis in GSE12021 (**A**) and GSE1919 (**B**) datasets. **C**, **D** Boxplot shows the differential expression of the six genes between healthy and OA groups in the GSE12021 (**C**) and GSE1919 (**D**) datasets. **E**, **F** Detailed diagnostic information (healthy/OA) and expression patterns of candidate genes between high-risk score and low-risk score groups in the GSE12021 (**E**) and GSE1919 (**F**) datasets, thus validating the diagnostic performance of the six-gene signature. Upper layer: risk score for each sample; middle layer: OA status (red: OA, green: healthy); lower layer: heatmap shows the gene expression

To verify the diagnostic gene signature’s reliability, we calculated the risk score of the samples in GSE12021 and GSE1919 using the same coefficients and validated the diagnostic ability of the gene signature (Fig. 4A,B shows the ROC curve for assessing the overall diagnostic performance of the risk score and gene expression; Fig. 4E,F shows the risk score in the upper layer, the OA status in the middle layer, and gene expression in the lower layer). Although the expressional differences of some genes (i.e., *MTHFD1*, *PDK4*, *POU4F1*, and *NCKAP1*) were not statistically significant (Fig. 4C,D), the basic changing trends were consistent with those in the original data (Fig. 3D). Notably, *TM9SF2* was significantly upregulated in the OA group among all datasets (Figs. 3D, 4C,F).

### Functional enrichment and cell specificity analyses for the signature gene, *TM9SF2*

**Fig. 5:**
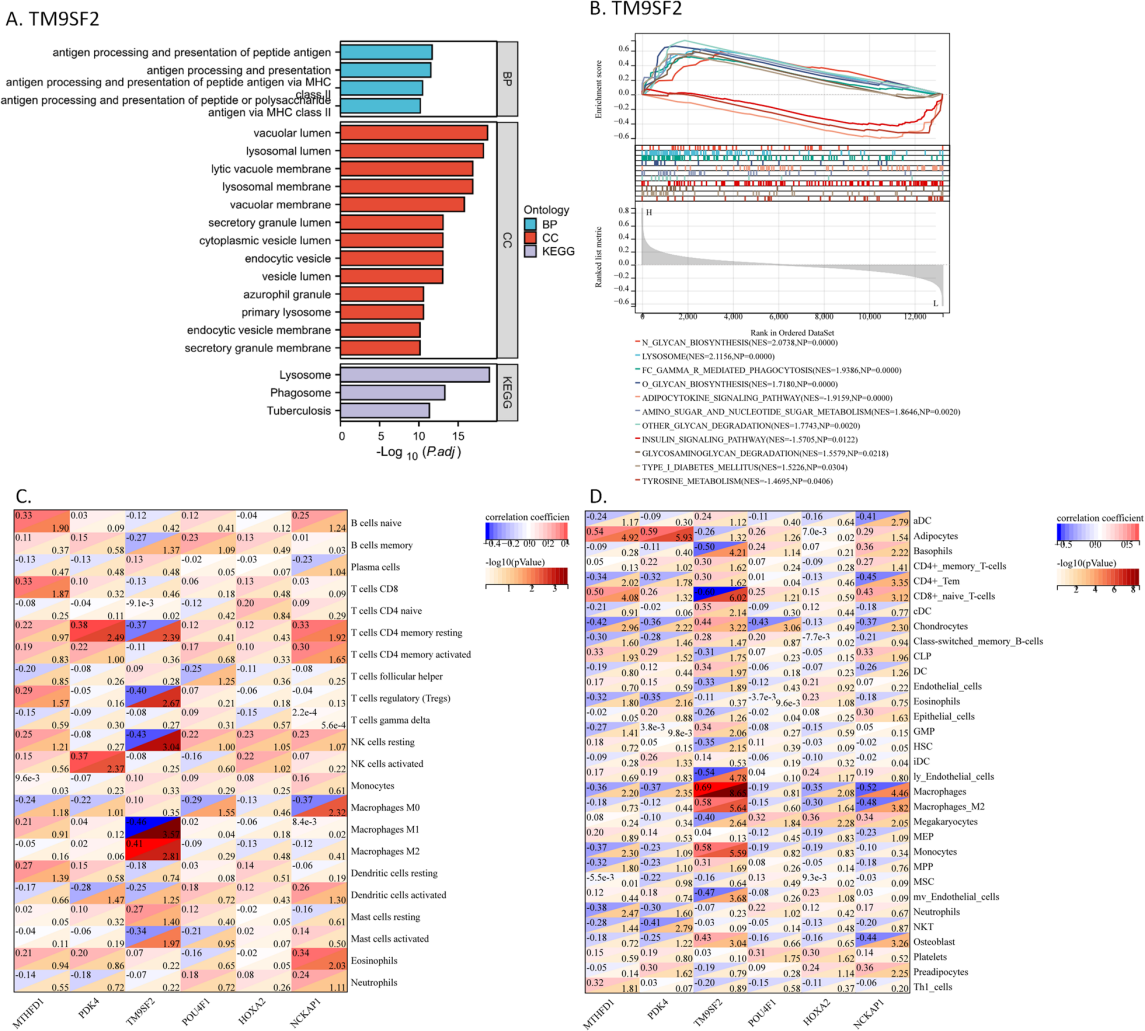
Functional enrichment analysis and cell-specificity analysis. **A** Functionally enriched annotations from GO and KEGG analyses for the positively correlated genes of TM9SF2, suggesting potential functions, including antigen processing and presentation, lysosome, and phagosome. **B** Enriched KEGG annotations in high- or low-*TM9SF2* expression samples after GSEA, suggesting potential functions, including lysosome, phagocytosis, and tyrosine metabolism. **C** The heatmap shows the correlation between the six genes and CIBERSORT-identified immune cells. There is a strong correlation between TM9SF1 and macrophages M1/M2. **D** The heatmap shows the correlation between the six genes and xCell-identified immune cells. There is a strong correlation between TM9SF1 and macrophage M2

To further understand the function of TM9SF2 in OA synovial tissue, we performed a correlation analysis for *TM9SF2* and GO/KEGG pathway enrichment analysis results for *TM9SF2* positively correlated genes. TM9SF2 was associated with phagosome, lysosome, and antigen processing/presentation via MHCII (Fig. 5A).

We performed a GSEA between *TM9SF2* high- and low-expression samples, classified based on the median expression of *TM9SF2*. The high-*TM9SF2* expression samples were enriched in the following KEGG pathways: “FC_GAMMA_MEDIATED_PHAGOCYTOSIS,” “LYSOSOME,” and “N/O_GLYCAN_BIOSYNTHESIS,” and the low *TM9SF2* samples were enriched in “TYROSINE_METABOLISM,” “INSULIN_SIGNALING_PATHWAY,” and “ADIPOCYTOKINE_SIGNALING_PATHWAY” (Fig. 5B). TM9SF2 was involved in the functions of phagocytosis, lysosome, and glycan biosynthesis, and could potentially inhibit tyrosine metabolism, insulin, and adipokine signaling pathways.

Many immune cells infiltrate the OA synovium, and we further studied the correlation between this gene and immune cells. From CIBERSORT analysis, *TM9SF2* was found to be positively correlated with macrophages M2 and negatively with macrophages M1 (Fig. 5C). After xCell analysis, TM9SF2 was found to be positively correlated with both macrophages and M2 macrophages (Fig. 5D).

### TM9SF2 regulates the phagocytic function of macrophages M2

**Fig. 6:**
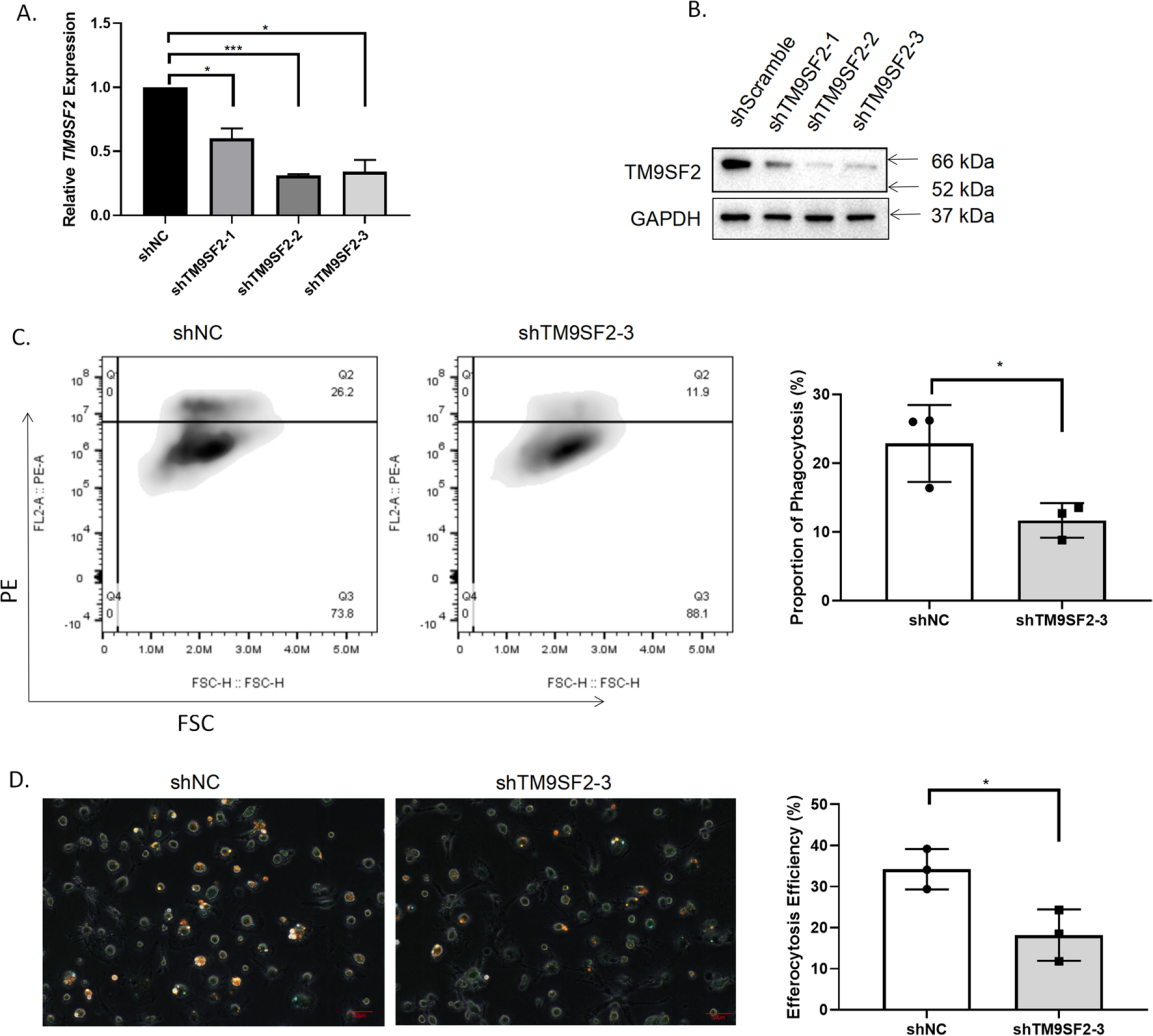
The effect of TM9SF2 on phagocytosis of macrophages M2. **A**, **B** Knocking down *TM9SF2* in THP-1-derived macrophages M2 at mRNA (**A**) and protein (**B**) levels. **C** The downregulation of macrophage phagocytosis on PE-stained IgG-coated latex beads by knocking down *TM9SF2*. **D** Downregulation of macrophage phagocytosis on PKH26-stained apoptotic Jurkat cells following *TM9SF2* knockdown. Flow cytometry and immunofluorescence results with statistical values obtained from three biological replicates in one technical replicate. The data are representative of three independent experiments. *P < 0.05, two-sided t-test. Red scale bar: 50 μm

According to the GESA and immune cell infiltration analyses described above, we speculated that upregulated *TM9SF2* expression potentially regulated the phagocytic function of macrophages M2 in OA. Therefore, we silenced *TM9SF2* by siRNA in THP-1-derived macrophage M2 (Fig. 6A,B) and cocultured them with IgG-coated beads to assess phagocytosis level. Knocking down *TM9SF2* reduced the proportion of phagocytic macrophages (Fig. 6C). Because apoptotic cells are accumulated in the synovium in OA, we tested efferocytosis efficiency by co-culturing macrophages M2 with PKH26-labeled apoptotic Jurkat cells. Knocking down *TM9SF2* reduced the efferocytosis of THP-1-derived macrophages M2 (Fig. 6D).

**Fig. 7:**
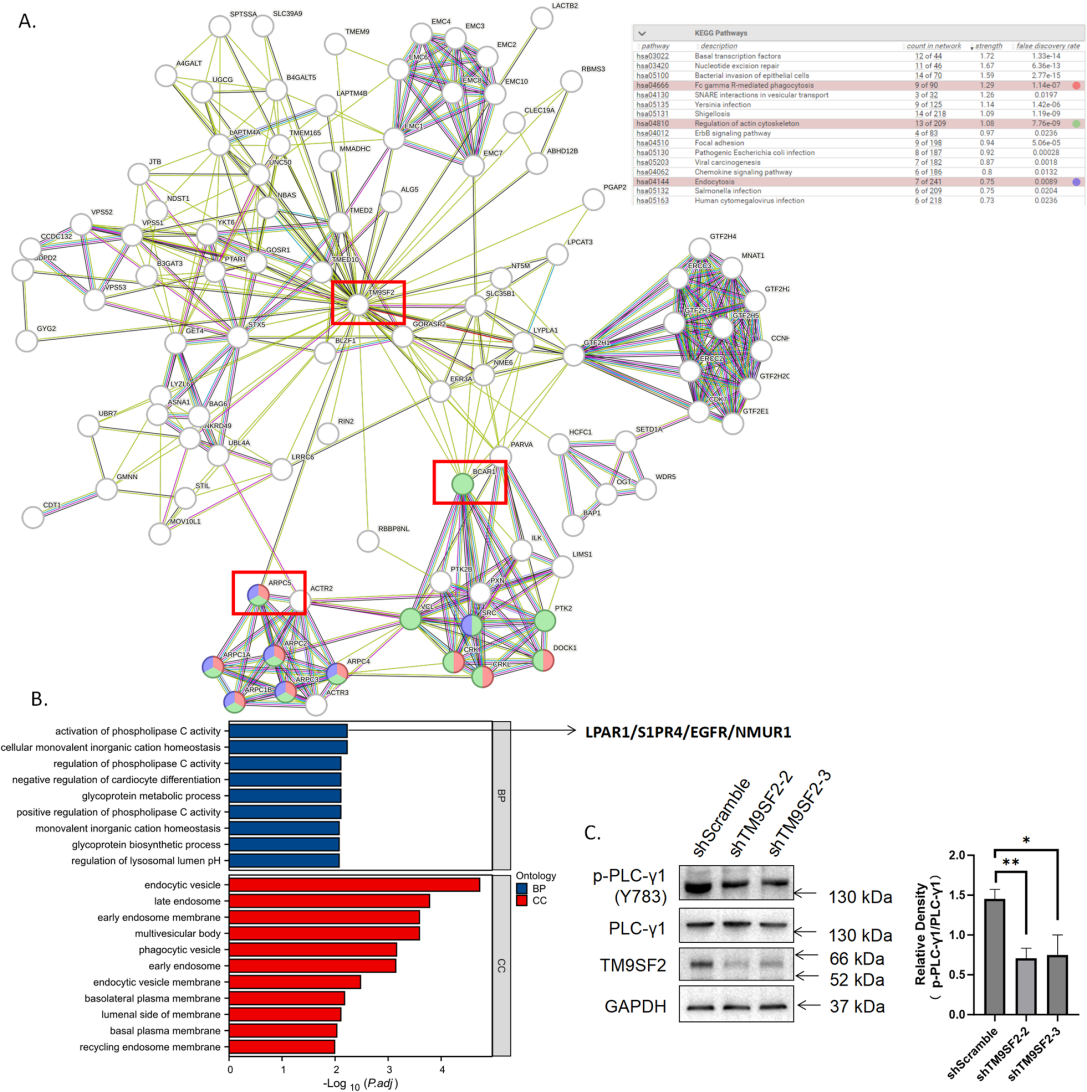
Proteins interacting with TM9SF2 and their enriched annotations. **A** The TM9SF2-centered STRING PPI network was enriched in functions “Fc gamma R-mediated phagocytosis,” “Regulation of actin cytoskeleton,” and “Endocytosis” as evidenced by the direct interaction with ARPC5 and BCAR1. **B** Proteins interacting with TM9SF2 (from BioGRID and HIPPIE databases) were enriched in functions of “activation of phospholipase C activity,” “endocytic vesicle,” and “phagocytic vesicles.” Enriched proteins are shown on the right side (pointed out by arrow), such as LPAR1 and RAB9A. **C** Knocking down TM9SF2 attenuates PLC-γ1 activation, as reflected by phosphorylation at its Y783 position. Data are representative of three independent experiments. *P < 0.05, **P < 0.01, two-sided *t* test

To explore the mechanisms underlying the downregulation of phagocytosis by shTM9SF2, we performed a PPI network analysis in STRING. TM9SF2 was involved in functions of “Fc gamma R-mediated phagocytosis,” “Regulation of actin cytoskeleton,” and “Endocytosis” by potential interaction with ARPC5 or BCAR1 (Fig. 7A). To identify protein interactors of TM9SF2 and their associated functions, we combined the interacting proteins of TM9SF2 from BioGRID and HIPPIE databases and performed GO/KEGG enrichment analyses. TM9SF2 may promote phagocytosis by regulating phospholipase C activity by interacting with LPAR1/S1PR4/EGFR/NMUR1 (Fig. 7B). Given the connection between PLC-γ1 activation, calcium release, and phagocytosis, we tested the status of PLC-γ1 activation after knocking down TM9SF2 and found downregulation of PLC-γ1 activity (Fig. 7C), as evidenced by the phosphorylation of PLC-γ1 at Y783.

## Discussion

The pathogenesis and early diagnosis of OA are interesting and challenging issues. Metabolic changes often yield biomarkers for early diagnosis, however, sulfur metabolism has not been analyzed in synovial tissues in OA. In this study, by analyzing the scores of sulfur metabolism-related genes in OA, we found reduced sulfur metabolism in OA synovial tissues and increased OXPHOS. The results suggest that impaired sulfur metabolism may play a role in OA pathogenesis. We obtained 10 differentially expressed sulfur metabolism-related genes, which could distinguish between OA and healthy synovial tissues following consensus cluster analysis. Sulfur metabolism-associated mechanical changes in OA-dominated class C2 included the upregulation of hematopoietic cell lineage, CAM, extracellular matrix, and vesicles, along with the downregulation of tyrosine metabolism, fatty acid degradation, glycolysis/gluconeogenesis, and lipoprotein particles. LASSO COX regression identified six signature genes, which could well distinguish OA samples in both the training (merged from GSE55235/GSE55457/GSE82107) and the verification (GSE12021 and GSE1919) datasets. Among the six genes, *TM9SF2* was upregulated in OA synovial tissues, suggesting strengthened activity of “FC_GAMMA_MEDIATED_PHAGOCYTOSIS,” “LYSOSOME,” and “N/O_GLYCAN_BIOSYNTHESIS,” and attenuated activity of “TYROSINE_METABOLISM,” “INSULIN_SIGNALING_PATHWAY,” and “ADIPOCYTOKINE_SIGNALING_PATHWAY” in osteoarthritic synovial tissues. Correlation analysis among immune cells showed that *TM9SF2* was expressed in macrophages M2.

MTHFD1 has trifunctional enzymatic activities and catalyzes one of three sequential reactions during the interconversion of 1-carbon derivatives of tetrahydrofolate, the substrates for methionine, thymidylate, and de novo purine syntheses [40]. PDK4 is localized in the matrix of the mitochondria and regulates glucose and fatty acid metabolism [41]. It inhibits the pyruvate dehydrogenase complex by phosphorylating one of its subunits, either PDHA1 and PDHA2, further downregulating aerobic respiration and inhibiting the formation of acetyl-coenzyme A from pyruvate. PDK4 can inhibit ferroptosis by blocking pyruvate dehydrogenase-dependent pyruvate oxidation in human pancreatic ductal carcinoma cells, indicating its ability of metabolic reprogramming [42]. Furthermore, it is a sensitive marker of increased fatty acid oxidation in multiple tissue types and cell types [43]. TM9SF2 plays a role in small molecule transport or can act as an ion channel [44]. It can promote cell adhesion and phagocytosis of eukaryotic phagocytes [45, 46]. It has also been implicated as an oncogene in colorectal cancer owing to its promotive effect on the cell cycle and OXPHOS [47]. In this study, we provide evidence of the potential link between TM9SF2 and antigen presentation/macrophage M2. Macrophage polarization is essential in the development of OA-associated synovitis [5, 48]. Functionally, macrophages can be categorized into three types, namely, unstimulated macrophages M0, proinflammatory macrophages M1, and anti-inflammatory macrophages M2. In addition to its anti-inflammatory effect, macrophage M2 plays an important role in clearing synovial apoptotic cells, which can attenuate OA’s progression [49]. POU4F1 belongs to the POU-IV class of neural transcription factors which regulate the expression of specific genes involved in differentiation and survival, including osteoclast/neuron differentiation and BCL2-promoted cell survival [50–53]. Recently, Pou4f1 was found to be expressed in kidney infiltrating macrophages in progressive renal fibrosis, with the proportion of Pou4f1^+^ macrophages being correlated with the degree of macrophage–myofibroblast transition in human kidney tissues [54]. TGF-β1 can promote the expression of neuronal differentiation marker, Tubb3, and neuron development transcription factor, Pou4f1, in bone marrow-derived macrophages [54, 55]. The link between POU4F1 and macrophages may partly explain the negative correlation between POU4F1 and lysosome in macrophages. HOXA2, as a transcription factor regulating gene expression during cell morphogenesis, cell differentiation, and embryonic development, may be involved in the placement of hindbrain segments in their proper locations along the anterior–posterior axis [56]. Hoxa2 can regulate palate development by inhibiting osteogenic differentiation of palatal mesenchyme [57]. NCKAP1 is part of the WAVE complex that regulates lamellipodia formation [58, 59] and has an important positive regulatory role on disulfidptosis [22]. Interestingly, NCKAP1 disruptive variants lead to a neurodevelopmental disorder [60]. HOXA2, POU4F1, and NCKAP1 are all associated with the development of the nervous system, suggesting their potential connection with pain in patients with OA.

TM9SF2 is a member of the transmembrane 9 superfamily and localizes to early endosomes in human cells, which may play a role in small molecule transport or act as an ion channel [47, 61, 62]. In addition to its role in promoting tumorigenesis and facilitating cell adhesion and phagocytosis of eukaryotic phagocytes [45–47], studies on the function of TM9SF2 and its association with OA are scarce. We tested and confirmed the sustaining effect of TM9SF2 on macrophage phagocytosis. The phagocytosis-sustaining effect of TM9SF2 was potentially due to the interaction between TM9SF2 and molecules associated with cytoskeleton/focal adhesion, phospholipase C activity, and vesicle formation. Notably, one study showed that high accumulation of apoptotic cells in the synovium of OA with the impaired efferocytosis ability of synovial macrophages [49]. Therefore, we hypothesized that TM9SF2 may attenuate the progression of OA. Why upregulated TM9SF2 expression in OA synovial tissues could not reverse impaired efferocytosis of synovial macrophages and the role of TM9SF2 in the phagocytosis/efferocytosis/antigen processing and presentation of synovial macrophages merit further exploration. Inhibiting PLC-γ1 exerts a protective effect on cartilage against OA [63, 64]. However, given the role of TM9SF2-activated PLC-γ1 in facilitating efferocytosis of macrophages, the inhibition of PLC-γ1 may promote the OA progression by impairing efferocytosis. The link between the TM9SF2/PLC-γ1 axis and OA progression merits further studies.

Some limitations of our study warrant consideration. First, we identified synovial OA biomarkers by using a small number of samples (27 healthy and 30 OA samples) by integrating GSE55235, GSE55457, and GSE82107 datasets, all of which were obtained from synovial tissues and are reliable GEO resources. We validated the result in two other datasets, GSE12021 (9 healthy and 10 OA samples) and GSE1919 (5 healthy and 5 OA samples) but the results were inferred from a sample size, and more studies are needed to validate our findings. Second, this study was based on bioinformatics analysis of the transcriptome of clinical samples, and in vivo studies or cohort studies are needed to validate the real diagnostic performance of these biomarkers. Moreover, the specific connection between the upregulated TM9SF2 expression and PLC-γ1 activation remains unclear. We think that it may be a result of the interaction between TM9SF2 and LPAR1/S1PR4/EGFR/NMUR1 and deserves further investigation. Nevertheless, our study has many advantages. Since the progression of OA is irreversible, late-stage OA can only be treated by joint replacement. Synovial tissues are more accessible by biopsy than by obtaining cartilage tissues during surgery. Furthermore, synovial samples have a closer pathological correlation with OA than peripheral blood samples. Therefore, identifying biomarkers of OA from synovial tissues can facilitate the early diagnosis and intervention of patients with OA.

In conclusion, we identified a six-gene signature for the diagnosis of OA and explored their correlated functions and immune cell infiltration state in OA synovial tissues. The availability of genomic biomarkers with diagnostic potential is invaluable for patients with OA for early detection and treatment.

### Supplementary Information

**Additional file 1.** Table 1. Gene lists for analysis.

## Data Availability

All data were downloaded from GEO database with accession IDs (https://www.ncbi.nlm.nih.gov/geo/query/acc.cgi?acc=): GSE55235, GSE55457, GSE82107, GSE12021, GSE1919, GSE152805.

## References

[1] Sharma L (2021). Osteoarthritis of the knee. N Engl J Med.

[2] Allen KD, Thoma LM, Golightly YM (2022). Epidemiology of osteoarthritis. Osteoarthr Cartil.

[3] Xu M, Ji Y (2023). Immunoregulation of synovial macrophages for the treatment of osteoarthritis. Open Life Sci.

[4] Mushenkova NV (2022). Phenotype diversity of macrophages in osteoarthritis: implications for development of macrophage modulating therapies. Int J Mol Sci.

[5] Wu CL (2020). The role of macrophages in osteoarthritis and cartilage repair. Osteoarthr Cartil.

[6] Kemble S, Croft AP (2021). Critical role of synovial tissue-resident macrophage and fibroblast subsets in the persistence of joint inflammation. Front Immunol.

[7] Chu CR (2012). Early diagnosis to enable early treatment of pre-osteoarthritis. Arthritis Res Ther.

[8] Kundu S (2020). Enabling early detection of osteoarthritis from presymptomatic cartilage texture maps via transport-based learning. Proc Natl Acad Sci U S A.

[9] Freyberg RH, Block WD, Fromer MF (1940). A study of sulfur metabolism and the effect of sulfur administration in chronic arthritis. J Clin Invest.

[10] Grimble RF (2006). The effects of sulfur amino acid intake on immune function in humans. J Nutr.

[11] Teigen LM (2019). Dietary factors in sulfur metabolism and pathogenesis of ulcerative colitis. Nutrients.

[12] Kim CJ (2009). L-cysteine supplementation attenuates local inflammation and restores gut homeostasis in a porcine model of colitis. Biochim Biophys Acta.

[13] Jain SK, Micinski D, Parsanathan R (2021). l-Cysteine stimulates the effect of vitamin D on inhibition of oxidative stress, IL-8, and MCP-1 secretion in high glucose treated monocytes. J Am Coll Nutr.

[14] Parpoudi S (2022). Effect of N-acetyl-L-cysteine on inflammation after intraperitoneal mesh placement in a potentially contaminated environment: an experimental study in the rat. Asian J Surg.

[15] Fonseca KM (2021). Anti-inflammatory effect of L-cysteine (a semi-essential amino acid) on 5-FU-induced oral mucositis in hamsters. Amino Acids.

[16] Yang J (2021). Targeting cell death: pyroptosis, ferroptosis, apoptosis and necroptosis in osteoarthritis. Front Cell Dev Biol.

[17] Liu S (2023). The role of regulated programmed cell death in osteoarthritis: from pathogenesis to therapy. Int J Mol Sci.

[18] Gasol E (2004). Membrane topology of system xc-light subunit reveals a re-entrant loop with substrate-restricted accessibility. J Biol Chem.

[19] Maschalidi S (2022). Targeting SLC7A11 improves efferocytosis by dendritic cells and wound healing in diabetes. Nature.

[20] Ye Y (2022). Repression of the antiporter SLC7A11/glutathione/glutathione peroxidase 4 axis drives ferroptosis of vascular smooth muscle cells to facilitate vascular calcification. Kidney Int.

[21] Koppula P, Zhuang L, Gan B (2021). Cystine transporter SLC7A11/xCT in cancer: ferroptosis, nutrient dependency, and cancer therapy. Protein Cell.

[22] Liu X (2023). Actin cytoskeleton vulnerability to disulfide stress mediates disulfidptosis. Nat Cell Biol.

[23] Ashburner M (2000). Gene ontology: tool for the unification of biology. Gene Ontol Consort Nat Genet.

[24] The Gene Ontology resource: enriching a GOld mine. Nucleic Acids Res. 2021;49(D1):D325-34. 10.1093/nar/gkaa1113 PMC7779012 33290552

[25] Davis S, Meltzer PS (2007). GEOquery: a bridge between the Gene Expression Omnibus (GEO) and BioConductor. Bioinformatics.

[26] Ritchie ME (2015). limma powers differential expression analyses for RNA-sequencing and microarray studies. Nucleic Acids Res.

[27] Hänzelmann S, Castelo R, Guinney J (2013). GSVA: gene set variation analysis for microarray and RNA-seq data. BMC Bioinform.

[28] Wickham H, Wickham H (2016). Data analysis. ggplot2: elegant graphics for data analysis.

[29] Wilkerson MD, Hayes DN (2010). ConsensusClusterPlus: a class discovery tool with confidence assessments and item tracking. Bioinformatics.

[30] Krijthe JH. Rtsne: T-distributed stochastic neighbor embedding using Barnes-Hut implementation. R package version 0.13. https://github.com/jkrijthe/Rtsne. 2015.

[31] Yu G (2012). clusterProfiler: an R package for comparing biological themes among gene clusters. OMICS.

[32] Liberzon A (2015). The molecular signatures database (MSigDB) hallmark gene set collection. Cell Syst.

[33] Subramanian A (2005). Gene set enrichment analysis: a knowledge-based approach for interpreting genome-wide expression profiles. Proc Natl Acad Sci U S A.

[34] Chen B (2018). Profiling tumor infiltrating immune cells with CIBERSORT. Methods Mol Biol.

[35] Aran D, Hu Z, Butte AJ (2017). xCell: digitally portraying the tissue cellular heterogeneity landscape. Genome Biol.

[36] Zeng D (2021). IOBR: multi-omics immuno-oncology biological research to decode tumor microenvironment and signatures. Front Immunol.

[37] Simon N (2011). Regularization paths for Cox's proportional hazards model via coordinate descent. J Stat Softw.

[38] Friedman J, Hastie T, Tibshirani R (2010). Regularization paths for generalized linear models via coordinate descent. J Stat Softw.

[39] Robin X (2011). pROC: an open-source package for R and S+ to analyze and compare ROC curves. BMC Bioinform.

[40] Christensen KE (2009). The MTHFD1 p.Arg653Gln variant alters enzyme function and increases risk for congenital heart defects. Hum Mutat.

[41] Rowles J (1996). Cloning and characterization of PDK4 on 7q21.3 encoding a fourth pyruvate dehydrogenase kinase isoenzyme in human. J Biol Chem.

[42] Song X (2021). PDK4 dictates metabolic resistance to ferroptosis by suppressing pyruvate oxidation and fatty acid synthesis. Cell Rep.

[43] Pettersen IKN (2019). Upregulated PDK4 expression is a sensitive marker of increased fatty acid oxidation. Mitochondrion.

[44] Schimmöller F (1998). Characterization of a 76 kDa endosomal, multispanning membrane protein that is highly conserved throughout evolution. Gene.

[45] Perrin J (2015). The nonaspanins TM9SF2 and TM9SF4 regulate the plasma membrane localization and signalling activity of the peptidoglycan recognition protein PGRP-LC in Drosophila. J Innate Immun.

[46] Bergeret E (2008). TM9SF4 is required for Drosophila cellular immunity via cell adhesion and phagocytosis. J Cell Sci.

[47] Clark CR (2018). Transposon mutagenesis screen in mice identifies TM9SF2 as a novel colorectal cancer oncogene. Sci Rep.

[48] Zhao K (2023). Effects of synovial macrophages in osteoarthritis. Front Immunol.

[49] Del Sordo L (2023). Impaired efferocytosis by synovial macrophages in patients with knee osteoarthritis. Arthritis Rheumatol.

[50] Huang L (2014). Pou4f1 and pou4f2 are dispensable for the long-term survival of adult retinal ganglion cells in mice. PLoS ONE.

[51] Liu L (2020). POU4F1 promotes the resistance of melanoma to BRAF inhibitors through MEK/ERK pathway activation and MITF up-regulation. Cell Death Dis.

[52] Schulze-Späte U (2007). Brn3 transcription factors control terminal osteoclastogenesis. J Cell Biochem.

[53] Zou M (2012). Brn3a/Pou4f1 regulates dorsal root ganglion sensory neuron specification and axonal projection into the spinal cord. Dev Biol.

[54] Tang PM (2020). Neural transcription factor Pou4f1 promotes renal fibrosis via macrophage-myofibroblast transition. Proc Natl Acad Sci U S A.

[55] Tang PC (2022). Single-cell RNA sequencing uncovers a neuron-like macrophage subset associated with cancer pain. Sci Adv.

[56] Seifert A (2015). Role of Hox genes in stem cell differentiation. World J Stem Cells.

[57] Iyyanar PPR, Nazarali AJ (2017). Hoxa2 inhibits bone morphogenetic protein signaling during osteogenic differentiation of the palatal mesenchyme. Front Physiol.

[58] Kitamura Y (1997). Interaction of Nck-associated protein 1 with activated GTP-binding protein Rac. Biochem J.

[59] Whitelaw JA (2020). The WAVE regulatory complex is required to balance protrusion and adhesion in migration. Cells.

[60] Guo H (2020). NCKAP1 disruptive variants lead to a neurodevelopmental disorder with core features of autism. Am J Hum Genet.

[61] Tanaka A (2017). Genome-wide screening uncovers the significance of N-sulfation of heparan sulfate as a host cell factor for chikungunya virus infection. J Virol.

[62] Li Q (2019). LINC01232 exerts oncogenic activities in pancreatic adenocarcinoma via regulation of TM9SF2. Cell Death Dis.

[63] Cai H (2018). The inhibition of PLCγ1 protects chondrocytes against osteoarthritis, implicating its binding to Akt. Oncotarget.

[64] Chen X (2021). PLCγ1 inhibition-driven autophagy of IL-1β-treated chondrocyte confers cartilage protection against osteoarthritis, involving AMPK, Erk and Akt. J Cell Mol Med.

